# mHealth Approaches in Managing Skin Cancer: Systematic Review of Evidence-Based Research Using Integrative Mapping

**DOI:** 10.2196/mhealth.8554

**Published:** 2018-08-02

**Authors:** Jihye Choi, Youngtae Cho, Hyekyung Woo

**Affiliations:** ^1^ Graduate School of Public Health Department of Public Health Science Seoul National University Seoul Republic Of Korea

**Keywords:** skin cancer, mHealth, e-Health, mobile technology, teledermatology, melanoma

## Abstract

**Background:**

mHealth, which encompasses mobile health technologies and interventions, is rapidly evolving in various medical specialties, and its impact is evident in oncology. In particular, mHealth has established itself as a prominent part of dermatology for cancer screening. Intensified research to seek its use and effectiveness in each phase of the skin cancer continuum is needed in this fast-growing field of teledermatology.

**Objective:**

The purpose of this review was to describe current trends in research addressing the integration of mHealth and its contributions across the skin cancer continuum.

**Methods:**

A systematic review framework was applied to the search using three electronic databases: PubMed, Web of Science, and Embase. We extensively reviewed appropriate studies regarding skin cancer and mobile technology published between 2007 and 2017. Studies of the role and impact of mobile technology in the prevention and management of skin cancer were included. We selected 18 studies adhering to the inclusion and exclusion criteria for analysis.

**Results:**

Of the 18 studies, 5 (28%) evaluated prevention interventions, 6 (33%) assessed diagnostic accuracy, and 7 (39%) pertained to feasibility in the context of mHealth approaches for skin cancer care. These studies portray the potential of mobile teledermatology in the prevention and management of skin cancer. However, not all phases of skin cancer involve mHealth, and not all have been addressed by research.

**Conclusions:**

This review extends our knowledge not only on the contributions of mHealth technologies, but also on their integration in different phases of skin cancer care. To optimize the effectiveness of mHealth in dermatology, larger numbers of robust, evidence-based studies on teledermatology implementations, distributed evenly across the care continuum, should be conducted so that research can be expanded to systematic reviews.

## Introduction

As mHealth, which includes mobile health technologies and interventions, has become more common in various medical fields, the concept has begun to receive more attention in cancer care [1]. In the last few years, mobile technologies have contributed to prevention, diagnosis, treatment, and follow-up in the field of skin cancer [2-4]. Skin cancer is a major health problem as it is one of the most common malignancies associated with significant morbidity. Despite the burgeoning interest in mHealth in the context of dermatological care, the outcomes of existing research on mobile technology in skin cancer care have been inconsistent [5,6].

The primary objective of this systematic review was to investigate recent research trends related to the use of mobile technology in the prevention and management of skin cancer, focusing on how such technology is evaluated and what impact it has in each phase across the cancer continuum. The review aims to answer the following key question: In what phase of the skin cancer continuum has mHealth technology been used and been effective among the adult population? We offer a holistic view and lessons for a roadmap of how mHealth technology has been engaged and its degree of success in the delivery of skin cancer care, setting the direction for future research.

## Methods

A systematic review was performed and reported in accordance with the Preferred Reporting Items for Systematic Reviews and Meta-Analyses (PRISMA) checklist. The detailed protocol was registered with PROSPERO (CRD 42018094442), an international prospective register of systematic reviews.

### Search Strategy and Information Sources

We searched PubMed, Web of Science, and Embase for articles published between January 1, 2007, and December 31, 2017. Appropriate studies addressing skin cancer and mobile technology were extensively reviewed. A list of relevant search terms was created around two domains: “skin cancer” and “mHealth.” The keywords—skin cancer, mHealth, e-Health, mobile technology, teledermatology, and melanoma—listed in no particular order, were included in the advanced search process using the conjunction “AND” and the disjunction “OR” as logical operators. An example of PubMed search strings is as follow: skin cancer and mHealth {“skin neoplasms”[MeSH Terms] OR (“skin”[All Fields] AND “neoplasms”[All Fields]) OR “skin neoplasms”[All Fields] OR (“skin”[All Fields] AND “cancer”[All Fields]) OR “skin cancer”[All Fields]} AND (“telemedicine”[MeSH Terms] OR “telemedicine”[All Fields] OR “mhealth”[All Fields]). Some of the equivocal terms were re-sorted into medical subject headings (MeSH), which brought forth more specific and relevant results. Upon obtaining various results according to the search criteria, we examined titles, abstracts, and keywords (MeSH terms) for further screening. Reference lists of selected studies were also checked for other potentially relevant studies.

### Eligibility Criteria

To be eligible for inclusion, records had to be official publications written in English and peer- reviewed articles in the scientific literature that constituted original research exclusive of systematic reviews. All publication dates had to be between 2007 and 2017, given that the use of mobile phones evolved and became widespread in the late 2000s, and more importantly, because the review intended to explore recent research trends. Records included in the review had to discuss the role and assess the effectiveness of mobile technology in all aspects of skin cancer interventions, ranging from prevention, feasibility and acceptability, and diagnostic accuracy to follow-up care. The population of interest was targeted to adults (aged 18 years or older). Excluded from the review were articles that were not original research, such as systematic reviews, correspondence letters, editorials, book chapters, briefing reports, articles that pertained to economic or cost analyses of teledermatology or app analyses, and articles describing studies in which no intervention had been performed. When the main theme of the intervention was not skin cancer, melanoma, or suspicious malignant lesions with a high likelihood of being cancerous, the study was excluded. Reports about dermatologic care pertaining to esthetics were also disregarded.

### Study Selection Process

The first task was to systematically search the three databases: PubMed, Web of Science, and Embase. When selecting the studies, we first performed a review of the titles and abstracts of all publications that were identified as relevant to this systematic review. Subsequently, duplicate citations across the databases were identified and removed using Endnote, and additional manual revision was performed for verification. Third, the remaining abstracts were meticulously checked for eligibility. Following this process, the full papers of the included abstracts were screened according to the inclusion and exclusion criteria. On the basis of the selection process, we were able to categorize the articles by the purpose of their interventions (ie, prevention, feasibility, diagnostic accuracy, and follow-up care) and the type of mobile technology used in the interventions.

### Data Collection and Extraction

Two authors (JC and YC) independently screened the titles and abstracts of all identified studies. Potentially relevant studies were retrieved in full text and further examined for eligibility by both authors. Disagreements were discussed and resolved with the corresponding author (HW). The first author (JC) extracted the following information into a synthesis table from the final set of relevant studies: author and publication date, setting/country, mHealth technology used in the intervention, description of target population (sample size, age, and comparison group), study objectives, study design and intervention content, outcome measures, and results.

### Quality Assessment for Risk of Bias

Quality assessments were performed to assess the methodological quality of included studies. Because this was a review of studies pertaining to more than one type of study design concerning different phases of the cancer continuum, the authors applied separate quality assessments accordingly. The authors used the Cochrane Collaboration tool [7-9] to make judgments about the extent of bias in each of the randomized controlled trial studies and to rate the information in each component of the paper. For diagnostic accuracy studies, the authors followed the revised version of Quality Assessment of Diagnostic Accuracy Studies [10,11] for quality assessment. As with the Cochrane Collaboration tool, the component ratings were scored as low risk, high risk, or unclear. Finally, the Newcastle-Ottawa Quality Assessment Form [12,13] was used for evaluating bias in cohort studies. The risk of bias for each of the studies was assessed by 2 authors (JC and YC). Any discrepancies between the authors were discussed with the corresponding author (HW) to reach consensus. The report of the risk of bias assessment is mentioned in the Results section, and a full presentation is included in Multimedia Appendix 1.

### Study Characteristics

Among the 18 articles selected for analysis, a considerable rise was observed in interest regarding mobile technology and skin cancer during the second half of the time period under consideration. Between 2007 and 2011, only five articles (28%) were published, whereas 13 articles (72%) were published between 2012 and 2017. The majority of the selected articles (14/18, 78%) were published in clinical dermatology journals and the remainder (4/18, 22%) in journals specializing in mHealth, medicine, preventive medicine, or photochemistry. Selected studies represented various geographical settings: Europe (9/18, 50%), the United States (4/18, 22%), Australia (3/18, 17%), Egypt (1/18, 6%), and Brazil (1/18, 6%). As for content, six studies (33%) assessed the accuracy of mobile technology in detecting and diagnosing skin cancer, seven studies (39%) examined the feasibility and acceptability of adopting mobile technology as well as its reported advantages in skin cancer management, and five studies (28%) concerned skin cancer prevention through mHealth interventions. A discussion of skin cancer follow-up via teledermatology was critically lacking among these articles. The findings are summarized in Multimedia Appendix 2.

## Results

### Search Results

**Figure 1:**
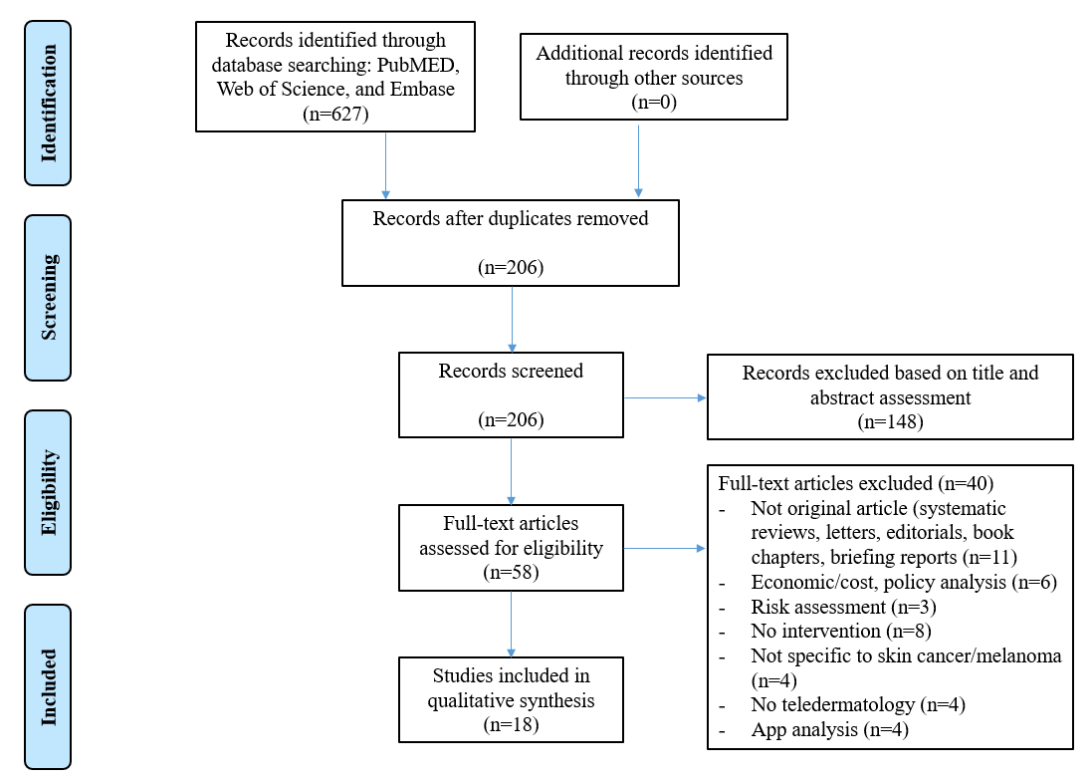
Flowchart of the systematic review process.

Figure 1 summarizes the literature search and review process. The foremost task was to systematically search PubMed, Web of Science, and Embase. A total of 627 records were retrieved, and duplicates within and among databases were removed, leaving a total of 206 records published between 2007 and 2017. These records were further screened by assessing whether the title or abstract contained the exact key terms (skin cancer, mHealth, e-Health, mobile technology, teledermatology, melanoma) and whether the content was in accordance with the established inclusion criteria. Following a detailed scrutiny of full-text articles for eligibility and exclusion of those that were inadequate for the analysis listed in the flowchart, the final set of records for the review comprised 18 studies. Regarding quality assessment, randomized controlled trial studies were assessed adhering to the Cochrane Collaboration tool. Of five potential biases (selection, performance, detection, attrition, and reporting), the most frequently occurring biases were performance and detection, whereas there was low risk of selection and attrition bias. Risk of bias for diagnostic accuracy studies that was assessed following Quality Assessment of Diagnostic Accuracy Studies was generally low with the exception of a few studies in which patients were not randomly or consecutively selected, index and reference tests were not blindly conducted, or loss to follow-up was observed. Finally, risk of bias was also generally low among the studies that were evaluated according to the Newcastle-Ottawa Quality Assessment Form, while risk of bias for some components could not be fully appraised due to insufficient report. An expanded report of the risk of bias assessment for each study can be found in Multimedia Appendix 1.

### Prevention

Skin cancer prevention efforts that entail mHealth approaches are often carried out via short message service (text messaging) and mobile apps as tools to facilitate sun practices [14-18]. In a study that used cellular text messaging as a reminder strategy to improve adherence to wearing sunscreen, participants who received text-message reminders were nearly twice as adherent to a regimen of daily sunscreen application compared with control participants not receiving reminders [14]. Another study showed that personalized educational emails and mobile messages can engender greater motivation and adherence to sunscreen use because these personalized messages conveyed knowledge about the advantages to the people targeted, in comparison to their counterparts who did not receive messages [15]. Positive changes in sun protection habits were seen when text messages addressed increasing self-efficacy, building behavioral capacity, or guiding outcome expectations. Persuasive messages with encouraging reminders about using skin protection from the sun and reducing any risk of skin cancer showed substantial compliance and improved skin self-examination among the recipients [16].

While significant improvement in behavior change was commonly observed in skin cancer prevention interventions via personalized, narrative text messages and emails, the success of a mobile phone app through which sun safety information was disseminated to promote sun protection practices has been insignificant and inconsistent [17,18]. Users of the app reported less time spent in the sun indicating some sun protection from the use of the app but no reduction in number of sunburns since the app was used, raising questions about the true effectiveness of the app [17]. Likewise, in a sequel study, although the app users reported a relatively higher mean percentage of time practicing all sun protection behaviors than in the previous study, the app appeared to confer weak improvement of sun protection, and the interventions were again unrelated to actual sunburn prevalence [18]. In these studies, individuals were generally inclined to download the mobile app, but once it was installed, the willingness of participants to continue using the app varied [17,18].

### Feasibility

The value of mHealth interventions in skin cancer management depends on successful transmission of medical data and dermatologic photographs without any technical issues and concerns being experienced by patients or health professionals [19,20]. The acceptance and feasibility of mobile teledermoscopy in the home environment were measured based on the ease of use, compatibility, and overall satisfaction perceived by melanoma patients [19]. While mobile teledermoscopy was well received and regarded as an easy process to conduct, concerns included trust in the telediagnosis and difficulty capturing some of the lesions, thereby indicating the need for more training to remedy competence issues among individuals, or for optimization of the technology [19]. Another study that investigated the feasibility of teleconsultation using a new generation of mobile phones with suspicious pigmented lesions demonstrated that mobile teledermatology has the potential become a flexible tool for enhanced self-monitoring for skin cancer screening [20].

Another prominent determinant of the feasibility of teledermatology was the attainment of high-quality images for all suspicious lesions likely to develop into cancerous lesions [21]. Transmission of images directly through mobile phone technology without the need to load them onto a computer enabled immediate analysis, making the process faster and more efficient [21]. Timeliness was an important criterion for determining the acceptance and feasibility of mHealth technology for the improved management of skin cancer. The expeditious analysis of high-quality dermoscopic images from patients referred by mobile phone teledermoscopy facilitated prioritization (faster response time from the designated dermatologist) and shorter waiting times prior to surgical treatment in comparison with the delayed arrival of paper referrals [22]. The feasibility of teledermatology for skin cancer was valid, particularly in the avoidance of unnecessary referrals for face-to-face consultations; that is, selecting only patients truly in need of dermatological intervention, in which case a definitive management decision was also established. This finding again aligns with those of other studies of the cost and time effectiveness of teledermatology [23]. The Breslow thickness, a measure that determines the stage of cancer, was on average significantly lower among patients managed by teledermatology than among their counterparts, and this clear difference between groups indicated the feasibility of teledermatology and its favorable impact in the initial prognosis of patients with melanoma [24]. In the case of Egyptian melanoma patients, the software-enabled mobile telephone with wireless connectivity was successful in both transmission and retrieval of diagnoses between onsite physicians and teleconsultants, demonstrating technical feasibility of using mobile teledermatology to expand access to dermatologic expertise and teaching where computers and Internet are absent [25].

### Diagnostic Accuracy

The diagnostic accuracy of mHealth technology or tele-evaluation for skin cancer remains a common theme to be substantiated in the discourse of teledermatology for skin [26]. Studies of the diagnostic accuracy of teledermatology have compared diagnoses made through teledermatology with those of in-person dermatologists or made by histology, which serve as the gold standard in current treatment. Four studies [26-29] reported a high accuracy of teledermatology in the primary diagnosis of skin cancer or lesions suspicious for malignancy. Diagnosis by the teledermatologist based on mobile phone-transmitted images was also in close agreement with that of the in-person dermatologist or histopathology used as the reference standard [27,28]. Likewise, equally high concordance was observed between photographs of lesions taken with a digital camera and the gold standard treatment, whether it was skin biopsy or evaluation by an in-person dermatologist [29]. The diagnoses of oncologists based on the direct visual inspection of electronically sent images were in close agreement (85.8% and 93.5%, respectively) with clinical descriptions and attached information, from which they were blinded [29].

In contrast, two studies [30,31] indicated that face-to-face diagnoses made by dermatologists were not in any way less accurate than diagnoses made by teledermatologists and that teledermatology was inferior to face-to-face dermatology. Borvë et al reported that primary face-to-face diagnoses made by dermatologists showed higher or similar, but not lower, accuracy than those made by teledermatologists who assessed incoming images from the mobile phone app used in the intervention. Accuracy then increased for all dermatologists when it came to distinguishing lesions as benign or malignant. Interobserver concordances between face-to-face dermatologists and teledermatologists, and among teledermatologists were virtually identical for all levels of diagnosis, with no particular overriding success among teledermatologists [30]. For clinical diagnosis, interobserver concordance of telediagnosis was lower than that of face-to-face diagnosis and the number of discordant telediagnoses increased with the progression of dermoscopic steps; clinical evaluation was superior for detailed diagnoses [31]. Occasional disagreement also occurred between the teledermatologist and the in-person dermatologist in the diagnosis of older patients after controlling for other variables [27].

## Discussion

### Principal Findings

We aimed to systematically review recent research trends in the integration of mHealth into the prevention and management of skin cancer by examining 18 studies found to be appropriate for a qualitative analysis. Teledermatology has gained popularity in the oncology community [32]. The emerging interest in the subject has called for intense scrutiny of mHealth interventions for skin cancer. With regard to skin cancer prevention, personalized text messaging as a reminder and informative tool successfully persuaded those at risk of skin cancer to practice sun protection behavior more conscientiously, which indicated that interventions incorporating text messages might be an effective innovative preventive health measure against the development of skin cancer [33]. The consensus has been that teledermatology targeting high-risk skin cancer patients is feasible and promising based on the positive responses and general willingness of the at-risk population to accept teledermatology, which is likely to persist given the continuing advancement of technological resources [34]. By contrast, opposing views exist about teledermatology in the diagnosis of skin cancer. Concerns and skepticism about underdiagnosis are evident due to previous failures to distinguish malignant tumors from benign ones and a high rate of discordance between teleconsultations and histological examinations [35]. The unprecedented merger of mobile technology and skin cancer management may still be in a nascent stage [36,37]. Continued research and numerous trials will be required to realize the potential for the expansion of interdisciplinary work in mHealth and skin cancer. Mobile teledermatology at this stage is perhaps best seen as a complementary diagnostic tool that aids clinicians, rather than as one that completely supplants in-person examinations causing omission of communication between doctor and patient [38].

Although mHealth technologies may not supersede conventional clinical procedures or human decision making, efforts have been made to establish mHealth as an overarching infrastructure to advance the process of skin cancer care [39]. The key mHealth technologies can be categorized as follows: mobile phone apps, text messaging, digital hand-held devices, and Web-based systems [40]. Each type of technology was proposed to target at least one phase in cancer care delivery to assist patients and medical professionals. However, differences in the role of mHealth technology between earlier and later phases of the cancer care continuum are noteworthy. Based on the categories mentioned above, the selected articles were regrouped according to how mHealth technologies were used to intervene in a specific phase of cancer care continuum. Table 1 shows that the application of mHealth technologies in skin cancer care continuum, and consequently the focus of current research, tend to be skewed toward store-and-forward diagnosis, with a few in the prevention and treatment phases. In particular, mobile phones with digital cameras or teledermascopes attached, along with the concomitant network-based communication system for relaying images, were the most frequently used technologies in the diagnosis phase of skin cancer management. We speculate that this is because mHealth has been largely used to support data collection and structured activities, such as automatic measurement and monitoring of patients’ vital signs during the progression of a disease [41].

In contrast, the treatment and follow-up phases of skin cancer care were least often addressed in the literature on teledermatology; no intervention was observed in the follow-up care of the continuum among the studies reviewed. The effectiveness of skin self-examination and the reduction of waiting times before surgery were discussed as part of the treatment phase [40], but evaluation of actual treatment procedures, including remote surgery with mobile technology intervention, has yet to occur. Although skin self-examination using mobile teledermatology is thought to effectively decrease unnecessary follow-up exams [41], its shortcomings should not go unremarked. For instance, skin self-examination using mobile teledermoscopy may be possible but may require assistance to photograph hard-to-see body areas [19,42].

Another distinct weakness of incorporating mobile technology in skin cancer management is that, given the nature of the store-and-forward technique, instantaneous feedback is limited because image analysis is essentially a human-executed, highly intricate task [43]. This process entails more than merely transmitting and processing numerical medical data; images need to be of high quality and punctiliously inspected by dermatology specialists [44]. Very few studies have attempted complete real-time decision making in teledermatology, probably due to the challenges and complexity in immediate image analysis relative to the current store-and-forward methodology [22]. These aforementioned limitations are often disregarded, perhaps due to a proclivity to highlight only the advantages of mHealth.

**Table 1:** Organization of studies: mHealth technologies used in the skin cancer care continuum.

mHealth Technology and study name		Cancer continuum							
		Prevention		Diagnosis/early detection					Treatment (primary management)/wait times
				Store and forward		Real-time			
**Text messaging**									
	Armstrong et al [14]	X							
	Szabó et al, 2015 [15]	X							
	Youl et al, 2015 [16]	X							
	Borvë et al, 2015 [22]							X	
	Ferrándiz et al, 2012 [24]							X	
**Mobile phone apps**									
	Buller et al, 2015 [17]	X							
	Buller et al, 2015 [18]	X							
	Horsham et al, 2016 [19]			X					
	Borvë et al, 2015 [22]			X				X	
	Lamel et al, 2011 [27]							X	
	Borvë et al, 2013 [30]							X	
**Hand-held digital devices (digital cameras attached to mobile phones, mobile dermascopes)**									
	Horsham et al, 2016 [19]			X					
	Massone et al, 2007 [20]			X					
	Hue et al, 2016 [21]	X				X			
	Borvë et al, 2015 [22]			X				X	
	Massone et al, 2014 [23]			X					
	Ferrándiz et al, 2012 [24]			X				X	
	Tran et al, 2010 [25]			X					
	Kroemer et al, 2011 [26]			X					
	Lamel et al, 2011 [27]			X				X	
	Markun et al, 2017 [28]	X		X				X	
	Silveira et al, 2014 [29]			X					
	Borvë et al, 2013 [30]			X					
	de Giorgi et al, 2016 [31]			X					
**Web-based (social networking sites, Skype, electronic referral system, virtual network)**									
	Massone et al, 2007 [20]			X					
	Hue et al, 2016 [21]	X				X			
	Borvë et al, 2015 [22]			X					
	Massone et al, 2014 [23]			X					
	Ferrándiz et al, 2012 [24]			X				X	
	Tran et al, 2010 [25]			X					
	Kroemer et al, 2011 [26]			X					
	Silveira et al, 2014 [29]			X					
	Borvë et al, 2013 [30]			X				X	
	de Giorgi et al, 2016 [31]			X					

Whether it is through mobile phone apps, text messaging, or digital photography functions attached to mobile phones, the potential is assumed to exist for mHealth technology to benefit cancer survivors based on various attested interventions that target the earlier phases of skin cancer. However, relevant literature on mHealth in follow-up care is scant, even for cancer in general, let alone skin cancer specifically [45,46]. The lack of studies on this subject casts doubt on the thoroughness and robustness of mHealth research in dermatology.

Therefore, we can be less sure of how mHealth technologies can influence the equally important posttreatment and recovery phases for cancer patients, who are at risk of recurrence at any point [47]. Unfortunately, the absence of research on follow-up care does not adhere to the intended aim of mHealth strategies for continuous health monitoring, leaving considerable doubt regarding the sustainability of mobile technology in skin cancer management [48,49].

The transition from acute cancer treatment to survivorship is often poorly managed, and skin cancer is no exception [50]. When designing and implementing mHealth-driven interventions targeting skin cancer survivors, the following key elements should be considered: tailored information and constant feedback from dermatology specialists, assistance with self-monitoring of suspicious lesions, and communication with other survivors through participation in social networks to sustain their well-being [51-53]. Mobile phone apps germane to health are the only mode of mHealth technology to have become recently available, although not always specifically for a given cancer type [54]. For the increasing population of cancer survivors with differing medical, psychosocial, and practical needs for daily living, mHealth apps could empower them by providing opportunities to engage in follow-up interventions that are informative, easily accessible, affordable, and personalized to their specific circumstances [55]. However, information on survivors is sparse, and very few apps have been formally tested. Doing so could be a tremendous step forward in widening the scope of effective melanoma follow-up care [56].

### Strengths and Limitations

This review extends our knowledge on the contributions and integration of mHealth in all phases of skin cancer care in order to gain a broad perspective on its uses and efficacy. Due to the number of available articles, this literature search was restricted to published articles from a limited number of selected sources. This may have led to selection and reporting bias in our review. Nevertheless, it serves as a good entry point from which readers can gain an overview of what mHealth technology has to offer in skin cancer care.

### Conclusion

The advent of mobile technology and its application are transforming the way health information is accessed and health care provided in various fields of medicine [57], including oncology. Accordingly, future mHealth interventions will need to be constantly revised and modernized [58]. To optimize the effectiveness of mHealth in skin cancer management, larger numbers of robust, evidence-based studies on teledermatology implementations should be conducted evenly across the cancer continuum from the mHealth perspective so that research can be expanded to systematic reviews.

## Appendix

Multimedia Appendix 1 Risk of bias assessment.

Multimedia Appendix 2 Summary of findings.
