# Supplementary material for: mHealth Approaches in Managing Skin Cancer: Systematic Review of Evidence-Based Research Using Integrative Mapping
Source: JMIR Mhealth Uhealth. 2018 Aug 2;6(8):e164. doi: 10.2196/mhealth.8554 (PMC6096168; doi:10.2196/mhealth.8554)
Supplement: Multimedia Appendix 1 [file mhealth_v6i8e164_app1.pdf]

Overview of quality assessment according to the Cochrane tool per study

Author	Risk of bias						
	Selection bias		Performance bias	Detection bias	Attrition bias	Reporting bias	Other bias
	Random sequence generation	Allocation concealment	Blinding of participants and personnel	Blinding of outcome assessment	Incomplete outcome data	Selective reporting	
Armstrong et al., 2009 [14]	Low	Low	High	High	Low	Unclear	
Szabo et al., 2015 [15]	Low	High	High	High	Low	Unclear	Systematic bias
Youl et al., 2014 [16]	Low	Low	High	Low	Low	Unclear	
Buller et al., 2015a [17]	Low	Unclear	High	High	Low	Unclear	
Buller et al., 2015b [18]	Low	High	High	High	Low	Unclear	Response bias

For randomized controlled trial studies pertaining to prevention in the cancer continuum, a total of five potential biases were assessed adhering to the Cochrane Collaboration tool: selection, performance, detection, attrition, reporting. If found, other biases were also indicated. The component ratings were scored as low risk, high risk or unclear. Under selection bias, all six studies were at low risk for random sequence generation but complete allocation concealment was exhibited in only one study, in which randomization sequences were concealed within numbered envelopes until interventions were assigned (Armstrong et al., 2009). Buller et al., 2015 did not clearly mention whether or how the allocation of the selected participants was concealed and the rest of the studies presented a high risk of bias for allocation concealment. Likewise, blinding of outcome assessment was an area of weakness in all of the studies although one study (Youl et al. 2014) managed to reduce detection bias as the interviewers were blinded to the participants' group allocation. In contrast, attrition bias was minimized in all studies as their outcomes were free of much loss to follow-up or almost none. Two studies, Buller et al. 2015b; Szabo et al. 2015, acknowledged that biases other than specified by the Cochrane tool, which were systematic bias, recall and social desirability bias and response bias.

Overview of quality assessment according to the QUADAS-2 tool per study

Author	Risk of bias				Concerns about applicability		
	Patient selection	Index test	Reference test	Flow and timing	Patient selection	Index test	Reference test
Massone et al., 2007 [20]	Low	Low	Low	Unclear	Low	Low	Low
Hue et al., 2016 [21]	High	Low	Low	Low	Low	Low	Low
Massone et al., 2014 [23]	Unclear	Low	Unclear	High	Low	Low	Low
Tran et al., 2010 [25]	Low	Low	Low	Unclear	Low	Low	Low
Kroemer et al., 2011 [26]	High	High	High	Unclear	Low	Low	Low
Lamel et al., 2011 [27]	Low	Low	Low	High	Low	Low	Low
Markun et al., 2017 [28]	Low	Low	Low	Unclear	Low	Low	Low
Silveira et al., 2014 [29]	Unclear	Low	Low	Low	Low	Low	Low
Borve et al., 2013 [30]	Low	Low	Low	Low	Low	Low	Low
de Giorgi et al., 2016 [31]	Low	Low	Low	Low	Low	Low	Low

For diagnostic accuracy studies, QUADAS-2 was applied to assess risk of bias with regards to the four domains:

patient selection, index text, reference test and flow and timing. The component ratings were also scored as low risk, high risk or unclear. Six out of ten studies included patients who were selected randomly or consecutively; patients and settings also appropriately matched the review question thereby lowering the risk of bias for patient selection. Kroemer et al., 2011 did not randomly select patients, but rather patients were self-referred to or referred by a local doctor for evaluation. Selection of target patients was somewhat narrow in Hue et al., 2016 and in the case of Silveria et al., 2014, it was not clearly explained. Next were assessments if the results of the index test (diagnostic test) and reference test (reference standard) were interpreted without the knowledge of each of the tests. In all studies, evaluations of index test and reference test were separately and blindly conducted thereby reducing the risk of verification bias. One exception was Kroemer et al., 2011, in which the same person reviewed, though separately, each set of clinical and dermoscopic; whether the dermatologist had knowledge of the results prior to each of the tests was not explained. Four out of seven studies scored as having low risk for patient flow. Participants in these studies received the same reference test (histopathological test), did not show significant dropouts in the analyses and the interval between the index and reference test was clearly indicated as being less than one month. In studies by Markun et al., 2017, Kroemer et al., 2011, Massone et al., 2007 and Tran et al., 2010, the interval between the index test and reference test or the dropout of the participants was not clearly stated as part of their methods. Unlike other studies, not all of the patients were included in the analysis in Lamel et al., 2011 and Massone et al., 2014 exhibited some loss at follow-up affecting the flow of participants whereas the size of loss to follow-up was unclear in Massone et al., 2007.

Overview of quality assessment according to the Newcastle-Ottawa Quality Assessment Form per study

Author	Selection*				Comparability of cohorts†	Outcome‡		
	Representativeness of exposed cohort	Selection of non-exposed cohort	Ascertainment of exposure	Outcome not present at baseline		Assessment of outcome	Sufficient follow-up duration	Adequate follow-up
Horsham et al., 2016 [19]	A	C	C	B	C	C	NR	B
Borve et al., 2015 [22]	A	A	A	A	A	E	A	B
Ferrandiz et al., 2012 [24]	A	A	A	A	A	D	NR	D

Abbreviation: NR, not reported

*Selection: 1) Representativeness of the exposed cohort: A, truly representative; B, somewhat representative; C, selected group; D, no description of the derivation of the cohort. 2) Selection of the non-exposed cohort: A, drawn from the same community as the exposed cohort; B, drawn from a difference source; C, no description of the derivation of the non-exposed cohort. 3) Ascertainment of exposure: A, secure record (e.g., surgical record); B, structured interview; C, written self-report; D, no description. 4) Demonstration that outcome of interest was not present at start of study: A, yes; B, no.

†Comparability of cohorts: 1) Comparability of cohorts on the basis of the design or analysis controlled for confounders: A, the study controls for age, sex, and marital status; B, study controls for other factors; C, cohorts are not comparable on the basis of the design or analysis controlled for confounders.

‡Outcome: 1) Assessment of outcome: A, independent blind assessment; B, record linkage; C, self-report; D, no description; E, other. 2) Was follow-up long enough for outcomes to occur? A, yes; B, no. 3) Adequacy of follow-up of cohorts: A, complete follow-up – all subjects accounted for; B, subjects lost to follow-up unlikely to introduce bias (number lost less than or equal to 20%); C, follow-up rate less than 80% and no description of those lost; D, no statement
