# Supplementary material for: mHealth Approaches in Managing Skin Cancer: Systematic Review of Evidence-Based Research Using Integrative Mapping
Source: JMIR Mhealth Uhealth. 2018 Aug 2;6(8):e164. doi: 10.2196/mhealth.8554 (PMC6096168; doi:10.2196/mhealth.8554)
Supplement: Multimedia Appendix 2 [file mhealth_v6i8e164_app2.pdf]

Study	Setting	mHealth Technology	Participants	Objectives	Study design/Intervention	Outcome measures	Results
Armstrong et al., 2009 [14]	USA	Mobile phone text messages	N=70<br>Aged 18 years or older<br>n=35 (control group with no text-message reminders)<br>n=35 (intervention group with text-message reminders)	To evaluate the effectiveness of cellular telephone text messaging as a reminder tool for improving adherence to sunscreen application. .	RCT; half of the participants received daily text-message reminders via cellular telephone for 6 weeks, and the other half did not receive reminders. The text-message reminders consisted of 2 components: a "hook" text detailing local weather information and a "prompt" text reminding users to apply sunscreen.	Adherence to sunscreen application measured by the number of days participants applied sunscreen during the 6-week study period.	The 35 participants who did not receive reminders had a mean daily adherence rate of 30/0% (95% confidence interval, 23.1%-36.9%). The 35 participants who received daily text message reminders had a mean daily adherence rate of 56.1% (95% confidence interval, 48.1%-64.1%). Among the participants in the reminder group, 69% reported that they would keep using the text-message reminders after the study, and 89% reported that they would recommend the text-message reminder system to others. Subgroup analysis did not reveal any significant demographic factors that predicted adherence.
Szabo et al., 2015 [15]	Hungary	Mobile phone text messages	N=149<br>(male=43,female=106)<br>Age: 18 years or older (mean age=36.94 years)<br>n=50 (group 1; no message)<br>n=50 (group2; no message)<br>n=49 (group 3 intervention group)	To improve sun protection habits of a sample population using cellular telephone text messaging and e-mails.	Non-blinded, RCT; messages were tailored to individuals, in the form of personalizing with greetings. One group received weekly electronic messages and two groups did not. Intervention group was sent 9 e-mail packages and 3 SMS messages.	Behavioral change; self-efficacy; adherence to sun-protection counselling; sun-exposure diary	Total motivation scores for adherence to sunscreen use improved (t=-1.954, p=0.054). Intervention group used sunscreens more often than the other groups according to their sun exposure diaries (F=8.713, p<0.05) and their interview results (F=3.44, p<0.05).
Youl et al., 2014 [16]	Queensland electoral roll or Medicare register, Australia	Mobile phone text messages	N=546 (male: 178 female: 368)<br>n=176 (skin self-examination),<br>n=187 (sun protection),<br>n=183 (attention control); population aged 18-42 years at high risk of skin cancer	To test the impact of a theory-based, text message-delivered behavioral intervention (Healthy Text) targeting sun projection or skin self-examination behaviors to attention control	RCT; Each of the three groups received 21 text messages about their assigned topic over 12 months (12 weekly messages for 3 months, then monthly messages for the next 9 months). Messages were personalized using participants' name and gender, skin cancer risk factors, number of times being burnt, previous performance of skin self-examination; telephone surveys	sun protection (habits, sunburn, sun tanning), skin self-examination outcomes, socio-demographic outcomes, behavior change, overall level of satisfaction with the intervention, importance of allocated health behaviors, and measures of engagement to the intervention	The sun protection (mean change 0.12; P=0.030) and skin self-examination groups (mean change 0.12; P=0.035) significantly greater improvement in their sun protection than the attention control group. The increase in the proportion of participants who reported any skin self-examination was significantly greater in the skin self-examination group (63%) than the sun protection (48%) or control groups (36%)

Buller et al., 2015a [17]	USA	Mobile application o n smartphones	N=202 (male= female=<br><br>A volunteer sample of adults aged 18 or order who owned an Android or iPhone smartphones	To evaluate a smartphone mobile application (Solar Cell) delivering real-time sun protection advice for a second time in a randomized trial	Pre-post RCT; for eight weeks, mobile application gave feedback on sun protection (sun safety practices and sunburn risk) and alerted users to apply/reapply sunscreen and get of the sun; displayed the hourly UV Index and vitamin D production based on the forecast UV Index, time, and location	Baseline, three- and eight-week post surveys assessed sun exposure and sun protection practices. Outcome measures included number of days practicing seven sun protection behaviors and days and minutes outdoors in the midday sun and number of sunburns in the past three months.	At 7-weeks, participants assigned to use the app reported wearing wide-brimmed hats when outdoors in the midday sun on a greater percentage of days than controls ($F=4.72$, $p=0.03$).<br>Males who used the app used less sunscreen lip balm than those no using the app ($F=4.19$, $p=0.04$).<br>Women using the app reported using more of all sun protection practices combined than those not using the app.<br>Educated participants, who used the app reported spending fewer days outdoors in the midday sun at the 7-week interim survey than controls ($F=3.55$, $p=0.03$).
Buller et al., 2015b [18]	USA	Mobile phone (Android) application software	N=604 (male=52.1%, female =47.9%)<br>Non-Hispanic and Hispanic adults aged 18 years or older.	To evaluate a smartphone mobile application (Solar Cell) delivering real-time sun protection advice for a second time in a randomized trial	Pre-post RCT with a ten-week follow-up; The mobile app provided advice on sun protection (protection practices and risk of sunburn) and alerts (to apply or reapply sunscreen and get out of the sun), hourly UV Index and vitamin D production based on the forecast UV Index, the phone's time and location, and user input.	Outcome measures included the number of days and number of hours spent in the sun, seven sun protection behaviors; sunburn prevalence was assessed with participants' experience of sunburn and the number of sunburns in the past 3 months.	Individuals using the app reported a larger mean percentage of time practicing all sun protection behaviors combined than nonusers. Participants not employed reported more days wearing wide-brimmed hats when using the app than those not using it ($F=8.57$, $p<0.01$).<br>Using the app was associated with reporting spending fewer hours outdoors in the midday sun by women than men ($F=4.88$, $p=0.03$)

Horsham et al., 2016 [19]	Australia	Apple iPhone 4 or 5 with dermascope attachment (FotoFinder Systems)	N=230 (all participants who completed pre-teledermoscopy survey n=49 (among the total, those who conducted mobile TD and completed post-TD survey. Aged 50-64 years at high risk of melanoma (fair skin or previous skin cancer)	To assess the self-reported consumer technology acceptance of mobile teledermoscopy when used for early melanoma detection during skin self-examination	Pre-post cohort study; a 27-item questionnaire completed pre-teledermoscopy. Post-teledermoscopy group, participants conducted a skin self-examination using mobile teledermoscopy in their homes for early melanoma detection. Submitted their dermoscopic and anatomical images to the study researchers and questionnaire via prepaid mail.	Perceived usefulness, perceived ease of use, attitude and intention, compatibility, facilitators, subjective norms, and trust. Post-teledermoscopy survey assessed the participants' satisfaction with mobile teledermoscopy, confidence conducting SSE alone or with mobile teledermoscopy and future use.	(Pre-teledermoscopy survey) 48% of items was rated as 'strongly agree' or 'agree' by 75% or more participants. Participants agreed in perceived ease of use, facilitators, subjective norms, attitude/intention and perceived usefulness. 86% agreed mobile TD motivated them to conduct SSE regularly also agreed with the ease of dermatoscope. 78% would use mobile TD again in the future. Barriers: photograph in a 'hard-to-see' body location (18%) and difficulty with photograph submission (35%).
Massone et al., 2007 [20]	Clinic setting, Austria	Built-in 2-megapixel camera (Sony Ericcson K 750i)	N=18 (male=12, female=6) Mean age=43, 38 years Median age=45 years	To investigate the feasibility to perform melanoma screening with both clinical and dermoscopic images acquired using a new generation of cellular phones.	Diagnostic accuracy method; one clinical image and one dermoscopic image without clinical data were sent to 2 teleconsultatns, who reviewed the cases independently and answered directly on the web application. Telediagnoses were compared with the face-to-face diagnosis which was taken as correct.	Diagnostic agreement (the concordance between the telediagnosis and the face-to-face diagnosis). Quality of each image (poor, fair, good, excellent)	The interobserver agreement among the two teleconsultants was 89% and 94% for the clinical and dermoscopic telediagnoses, respectively. Quality of clinical images judged poor for 31%, fair for 39%, good for 19%, and excellent for 4% of the cases. Dermoscopic images: 11% poor, 42% fair, 42% good, 5% excellent image quality.

Hue et al., 2016 [21]	Rural areas i n France	Mobile cellular phone (iPhone) teledermoscopy	N=289 (male=194, female=95); Aged 18 years or older agricultural population Skin lesions grouped into four management categories: no further treatment/follow-up required, follow-up at 12-month intervals, patient advised to make a dermatologist appointment, referral to a local dermatologist for a rapid race-to-face examination.	To assess the feasibility of real-time mobile teledermoscopic triage of a large number of agricultural workers by occupational physicians and medical officers	RCT; in a one-day teledermoscopic screening event, images of skin lesions and suspicious skin lesions were taken with a mobile cellular phones and subsequent dermoscopic images via the use of a polarized light contact dermoscope. Images were subsequently transferred anonymously via a website for teleconsultation on the dermatologist platform	Quality of both the clinical and dermoscopic images were first assessed. Feasibility was assessed based on the percentage of patients who were successfully evaluated by the teleconsultant and the percentage of patients with malignant melanocytic suspicions who were evaluated by a local dermatologist within ten days.	390 suspicious lesions were identified and 412 pictures generated among 199 patients (69%). 53% required no-follow-up. 8% of the patients were required to be followed-up at 12 month intervals. 31% were referred to a dermatologist for nonemergency appointments. 9% were referred to a local dermatologist for rapid examinations with 12 cases of suspected malignant melanocytic lesion.
Borve et al., 2015 [22]	One urban and one rural hospital settings Sweden	Smartphone (iPhone) application and hand-held dermoscope compatible with the smartphone (FotoFinder Handyscope)	N=816 eligible teledermoscopy (TDS) referrals from n=772 patients (cases; male=298, female=474, mean age, 54 years, range, 18-93 years) and 746 patients referred with paper referrals (controls; male=320, female=426, mean age, 61 years, range, 18-97 years)	To investigate whether smartphone teledermoscopic referrals could provide faster management of patients with skin cancer and more accurate prioritization of patients with skin lesions of concern compared to normal paper referrals without images	One-year case-control, prospective observational study; the general physician takes one clinical and one dermoscopic image with relevant clinical information. Upon sending the referral, the data is embedded on a secure web-based TDS platform. Simultaneously dermatologists receive an e-mail that a new referral is ready for assessment. The dermatologists log on to the platform to review the referrals on a display monitor and chooses from standardized triage responses	Outcome measures the final clinical or histopathological diagnosis of the referral lesions, the primary therapy applied, the number of visits needed, the waiting times from referral to diagnosis and/or treatment and any incidental findings.	Dermatologist's response in a mean time of 233 min (3.9h). Fastest response was 2 min and the slowest 46h. 79 referrals (98%) assessed in 24h. Paper referrals arrived after a mean time of 5 days (0-82 days). TDS group: requiring surgery with a diagnosis of malignant melanoma shorter waiting time for a first visit with a dermatologist ($p<0.0001$). Shorter waiting times to receive surgical treatment in the TDS group ($P<0.0001$). Median waiting time of 36 days for diagnosis and treatment for malignant lesion patients for TDS referrals and 85 days for paper-based referrals ($p<0.0001$).

Massone et al., 2014 [23]	Austria	Canon Powershot digital camera and a polarized light contact dermoscope (DermaLite Photo)	N=690 patients (male=642, female=48) Mean age = 47 years, range, 18-84 years.<br><br>n=962 dermoscopic images<br>n=123 clinical images from 962 lesions sent for teleconsultation	To investigate the feasibility of store-and-forward teledermatology triage system in a large number of patients	Images correlated by only age, sex and location of the lesion were transferred for teleconsultation via an already well-established virtual private network to two dermatologists (teleconsultants) for diagnosis and decision-making. Teleconsultants answered in 48h. A store-and-forward TD facility was used to make diagnosis and management of equivocal skin lesion; the TD facility was used as a triage system for skin cancers.	Image quality assessed using 3-point scale Lesions grouped into four diagnostic categories (Benign melanocytic, malignant melanocytic, benign non-melanocytic and malignant non-melanocytic skin lesions). Gold standards were used to assess teleconsultants' diagnostic accuracy.	Teleconsultants judged 88% (851/962) dermoscopic vs. 77% (95/123) clinical images of excellent quality, 10% (94/962) vs. 19% (23/123) of moderate and 1% (14/962) vs. 4% (5/123) of low image quality. Telediagnosis results were 78% benign melanocytic, 0.6% malignant melanocytic, 19% benign non-melanocytic and 2% malignant non-melanocytic skin tumors.<br>Strong concordance for remote evaluations with the gold standards in differentiation between benign from malignant skin lesions. 94% of lesions correctly classified by the teleconsultants
Ferrándiz et al., 2012 [24]	Hospital setting, Spain	Digital photo cameras (Coolpix 4300, Nikon or Cybershot DSC-W30 or DSC-W300, Sony)	N=201 (patients with primary cutaneous melanoma), n=67 (TD group, mean age 56.9 years), n=134 (non-TD group, mean age, 57.8) Aged 18 years or older	To evaluate differences in the initial prognosis of patients with cutaneous melanoma managed by teledermatology (TD) vs. other non-TD referral systems	Descriptive and longitudinal study; photographs and clinical information were uploaded and submitted via e-mail attachment to the public health service intranet. Following the teleconsultation, a report with the possible diagnosis and the referral decision to the Melanoma Clinic is returned to the primary care center.	Decisions on the referral of patients with suspicious skin lesions by store-and-forward TD vs. by a conventional referral system. Breslow thickness and tumor stage were recorded in each study group (TD and non-TD) and were compared.	The frequency of melanoma with a favorable initial prognosis was significantly higher in the TD group (70.1% vs 56.9%, P=0.03). The odds ratio of having a cutaneous melanoma with a favorable initial prognosis in the TD group was 1.96 (95% CI, 1.14-3.50; P=0.04)

Tran et al., 2010 [25]	Egypt	Sotware-enabled mobile telephone containing a 5-megapixel camera	N=30	To demonstrate that mobile telephones may be used on the African continent to submit both patient history and clinical photographs wirelessly to remote expert dermatologists, and to assess whether these data are diagnostically reliable.	The diagnoses formulated through face-to-face examination were tallied and compared with the diagnoses formulated through teleconsultation and diagnostic concordance rates were tabulated.	Feasibility was assessed based on diagnostic agreement between face-to-face consultation and local dermatologists' independent evaluation by teleconsultation	Diagnostic agreement between face-to-face consultation and the two local senior dermatologist performing independent evaluation by teleconsultation was achieved in 23/30 (70%) and in 22/30 (73%) cases, respectively (global mean 75%).<br><br>Mobile teledermatology is regarded as a technically feasible and diagnostically reliable method of amplifying access to dermatologic expertise.
Kroemer et al., 2011 [26]	Outpatient dermatology clinic, Austria	Mobile phone camera (Nokia N73 with a built-in 3.2-megapixel camera)	N=88 (males and females with benign and/or malignant skin tumors of melanocytic or nonmelanocytic origin)<br><br>A total of 322 clinical and 278 dermoscopic images of 113 skin tumors	To evaluate the diagnostic accuracy of clinical and dermoscopic image tele-evaluation for mobile skin tumor screening.	Over a 3-month period up to three clinical and dermoscopic images were obtained of 113 skin tumors from 88 patients using a mobile phone camera. Clinical and dermoscopic images of each lesion together with clinical information were separately transmitted for decision-making. The lesions were grouped into four diagnostic categories, and diagnoses were then compared with the gold standard.	Diagnostic concordance (results were compared with those obtained by face-to-face examination and histopathology as the gold standard).	Clinical and dermoscopic tele-evaluations demonstrated strong concordance with the gold standard (k=0.84 for each) and similar high sensitivity and specificity for all diagnostic categories. Clinical image tele-evaluation was superior to teledermoscopy.
Lamel et al., 2012 [27]	Skin cancer screening event, USA	Cellular phone (Google Android G1) with ClickDerm, a mobile-phone application to facilitate remote diagnosis of skin conditions by dermatologists.	N=86 participants with 137 skin lesions (male=35, female=50)<br>Mean age=45.24 years	To determine diagnostic and management concordance between in-person and teledermatology evaluations for patients at skin cancer screening whose clinical images and history were transmitted through mobile phones	Patients' clinical history and skin images captured by a software-enabled mobile phone. Patients assessed separately by an in-person dermatologist and a teledermatologist, who evaluated the mobile phone-transmitted history and images.	Primary outcomes measure: measurement concordance.<br><br>Secondary outcomes: aggregated diagnostic concordance and primary categorical diagnostic concordance.	Primary categorical diagnostic concordance between the in-person dermatologist and the TD: 82% (95% CI, 0.73-0.89; kappa 0.62); good agreement. Aggregated diagnostic concordance: 62% (95% CI, 0.51-0.71; kappa 0.60); good agreement.<br>Management concordance: 81%, (95% CI, 0.72-0.88; kappa 0.57); moderate agreement.

Markun et al., 2017 [28]	Hospital setting, Switzerland	5-megapixel camera of an iPod (Apple Inc.) touch fifth generation, Handyscope (Fotofinder)	N=188 patients (male=75, female=113)<br>Mean age=40.4<br><br>195 eligible skin lesions.	To estimate the diagnostic performance (sensitivity, specificity, and predictive values) of lesion-directed mobile teledermatology with or without dermoscopic imaging as a skin cancer screening intervention in a population representing a low-prevalence setting	Prospective diagnostic study; Images arranged by skin lesions were organized in 2 packages, the first one macroscopic images only and the second one with additional teledermascopic images, forwarded to the teledermatologist. TD first decided adequacy of images to allow assessment for cancer screening. In adequate cases, recommendations were given in 3 categories.	Test performance measures of teledermatology with and without teledermoscopy. Practicability of the techniques measured by skin lesions suitable for cancer screening, an estimate of potential value of the screening intervention to detect 1 case of skin cancer	Conventional TD: 100% sensitivity, 76.6% specificity, 14.6% positive predictive value, and 100% negative predictive value. Rule-out follow-up in 73.6% (134/182). Overall concordance with reference standard was 77.5% (k=0.20). Additional TD with conventional TD: 100% sensitivity, 84.9% specificity, 20.0% positive predictive value, and 100% negative predictive value. Rule-out follow-up in 81.8% (157/192). Overall concordance rate of 85.4% (k=0.29).
Silveira et al., 2014 [29]	Hospital setting, Brazil	Digital camera (Sony Cybershot DSC-5780, 8.1-megapixel resolution)		To evaluate the performance and accuracy of digital imaging in diagnosing skin cancer in remote areas of Brazil.	Digital images of suspicious lesions were electronically sent to two oncologists who blindly evaluated the images and provided a diagnosis (benign or malignant).	Overall accuracy (correct classification rate), sensitivity, specificity and predictive value. A skin biopsy was the gold standard.	Oncologist #1 classified 59 lesions as benign with the digital images; oncologist #2 classified 27 lesions as benign using the same images. The absolute agreement rates with direct visual inspection were 85.8% for oncologist #1 (95% CI, 77.1-95.2) and 93.5% for oncologist #2 (95% CI, 84.5-100.0).
Borvë et al., 2013 [30]	Hospital setting, Sweden	Smartphone (iPhone 4); a dermoscope attached to the smartphone (FotoFinder Handyscope); web-based TD platform (Tele-Dermis, iDoc24 AB); iPhone app (iDoc24 AB)	N=62 (male=38, female=24)<br>Mean age=64 years (range, 25-94 years)	To study diagnostic accuracy of mobile teledermoscopy solution; to determine the interobserver concordance between teledermascopists (TDs) and a dermatologist meeting the patient face-to-face; to assess the adequacy of the TD's management decisions; to evaluate the image quality obtained	During a 16-week period, patients with one or more suspicious skin lesions in need of a biopsy or excision included; the smartphone app used to send a clinical image (dermoscopy image and clinical information) to Internet platform. Two TDs assessed the incoming cases, providing a specific primary diagnosis and a management decision. Histopathological diagnosis was used as the gold standard.	Diagnostic accuracy of the face-to-face (FTF) dermatologist and TDs; interobserver concordances; image quality; level of diagnostic difficulty.	69 lesions included; Diagnostic accuracy: 66.7% (FTF), 50.7% (TD 1), 60.9% (TD 2).<br>Moderate to substantial agreement between TFT dermatologist and the TDs and between the TDs for interobserver concordances.<br>The TDs provided adequate management decisions for 98.6% and 100% lesions, respectively.<br>Image quality rated as excellent or sufficient in 94% and 84% of the cases by the respective TDs.

De Giorgi et al., 2016 [31]	Italy	Digital camera (Olympus Digital E-520, 7.1 Megapixel); hand-held dermatoscope (Heine Delta 20); Dermaphot (Heine Optotechnick)	N=10 (male=4, female=6) Mean age=51.3 (range, 26-69 years)	To determine the diagnostic concordance between the conventional face-to-face diagnosis and the telediagnosis of 10 dermatologists with expertise in dermato-oncology of 10 challengeing pigmented lesions.	Clinical and dermoscopic digital images of all selected lesions transmitted via e-mail to 10 dermatologists using a store-and-forward teledermatology method. Dermatologists provided telediagnoses with a step-by-step approach.	Diagnostic concordance; final histopathological diagnosis was considered the gold standard for comparison with a face-to-face and teledermatology diagnoses in statistical analysis.	Face-to-face results indicated moderate agreement between clinical and histopathological diagnoses (k=0.6); interobserver concordance of telediagnosis lower than face-to-face diagnosis (k=0.52); the concordance declined further (k=0.38) after the second dermoscopy step.
